# Correcting the Whole Error of Refraction and the Necessity for the Use of a Mydriatic

**Published:** 1889-08

**Authors:** R. O. Cotter

**Affiliations:** Macon, Ga.


### CORRECTING THE WHOLE ERROR OF REFRACTION AND THE NECESSITY FOR THE USE OF A MYDRIATIC.*

BY R. O. COTTER, M. D., MACON, GA.

While the specialty of Ophthalmology is yet in its early youth, this subject of the correction of errors of refraction, if not actually in its swaddling clothes, is at least a *vexata questio.* It is remarkable how teachers of Ophthalmology differ point-blank in their views concerning the matter of fitting glasses for ametropia. Some will claim that it is possible, by means of the opthalmoscope alone, to measure refraction without paralyzing the accommodation. Others admit the necessity for the use of the mydriatic, but many of these disagree as to how much, or if the total error should be corrected. It seems hardly necessary to argue the impossibility of getting at, by means of the ophthalmoscope alone, the true state of the refraction of an eye, whose ever varying muscle of accommodation must undoubtedly vitiate any attempt to measure its refraction. Of course I do not mean that we should

*Read before the Medical Association of Georgia, at Macon, April, 1889.

not verify (after the use of atropine) our work with the ophthalmoscope. To leave it aside we would be liable to make serious errors, and possibly overlook amblyopia, etc. So thoroughly am I convinced of the absolute necessity of using a mydriatic, believing as I do that there is without its use such a serious element of doubt about this most difficult and delicate work, that I have adopted its employment as my rule, invariable and positive. As to fitting the whole error—if a patient's total hypermetrophia is 1-20, why should we give him a glass which corrects only 1-36, 1-48, or less. Common sense ought to dictate that when we have to deal with an ametropic eye, we should ascertain (by completely paralyzing the accommodation) just what the total error is. Then we should, with a fully correcting glass, bring the eye up to a condition of normal refraction (emmetropia); then the ciliary muscle will have just the normal amount of focusing to perform. I use a four-grain to the ounce solution, and drop it in the eye sufficiently often to make the muscle thoroughly passive. If I had no other reason, there is one special class of cases in which I would especially insist upon the free use of atropine. These are the young hypermetropes who, from excessive use of their eyes, have brought on spasm of the ciliary muscle. When their vision is tested they have apparent myopia. They will accept—(concave) glasses, when if we paralyze their accommodation they will require + (convex) glasses. If their parents object to the use of glasses, or we, for any reason, think it advisable to not prescribe glasses for them, there is no better treatment than the enforced rest which thorough atropinization brings about for the tired and irritated muscle.

I am more and more convinced, by daily observation, that very many cases of real myopia begin in this way among children, who are hypermetropic. Their myopia, *apparent* only at first, has, by over use of the eye and ciliary spasm, become a *real* myopia at last. At the present, I am rather opposed to prescribing glasses for young *near-sighted* persons, yet I feel that the oculist, who does not do all he can to call the attention of the profession to the very great importance of having many of the errors of refraction in young persons scientifically corrected, does not do his duty. Many a young person with normal eyes, except for some easily remediable error of focus, is handicapped and pushed to the wall in the battle of life.

Of the very many cases in which the whole error was corrected, I have kept up very carefully with some seventy or eighty. In these seventy or eighty, I have only in two cases found it necessary to afterwards modify the strength of the glass. The following cases are perfectly fair samples of those to be found in my case book. As a *rule* I find it best to fit the whole error, though of course there *are* cases, for instance, where the patient's range of accommodation is considerable, where common sense plainly indicates a modification of the rule.

*Case 1.*—Miss E., age 16. Her eyes ache severely whenever she studies. Conjunctivæ, congested and suffused. Retinæ congested—not as a disease *per se*, however, but simply as a symptom. Her mother had about decided that it was useless for her to attempt to continue her studies. Vision appears as if she were myopic. Right=20–40, L.=20–40, she accepts—cylindrical glasses, axis at 180°. Under atropia the true condition is shown up. She had spasm of the ciliary muscle, with atropia. R. vis.=20–70, L.v.=20–50, R. and L. w. + 1–60 ⌒ cyl. + 1-72 axis 90°= 20–20, and with these glasses for close use she had in two weeks' time sufficiently recovered and resumed her studies. She is now pursuing a very severe curriculum of study in a northern seminary in perfect comfort.

*Case 2.*—Mr. W., (book-keeper) age twenty-three. Increase of his duties lately has brought on all of the symptoms described in Case 1, only to a very much severer degree. He cannot hold his eyes open even, from photophobia. It took about ten or twelve days' careful treatment and absolute rest of his eyes to get him ready to have his refraction tested. Vision before use of atropia, R=20–20, L=20–15, nearly. With atropia, R. and L.=20–40; R. and L. w. + 1-40=20–15. With these glasses he almost immediately secured perfect comfort. Of course, he only needs glasses for close work ; but with them for distance even, he reads 20–15.

*Case 3.*—Miss M., age, twenty-five. Health delicate, evidently from lack of out-door exercise; vision, R. and L. 20–30. Had very frequent and severe headaches, especially upon close use of her eyes. I could not, upon careful examination with the ophthalmoscope, discover at this time any congestion of either retina. As she exhibited some of the symptoms of astigmatism, I insisted upon testing her refraction. I found her whole error to be R. and L. + 1–60 $\bigcirc$ cyl. + 1–48, axis 90°. Vision with these, R. and L.=20–20. With these glasses for close use she gets a hundred-fold more comfort than she did before she was correctly fitted. She informs me also that she practices at the piano as much as she wishes to.

*Case 4.*—Mr. S., (book-keeper) age twenty-six. Has since childhood had an annoying inflammation of his eyelids, (blepharitis marginalis) and now it has grown so severe, unless he gets relief he fears he will lose his position. Vis. R. and L. 20–30. Greatly objects to the use of, or even trial of, glasses, but finally submits to the test. Under atropia, R. and L.=20–70, R. w. + 1–30 $\bigcirc$ cyl. + 1–60 ax. 90° 20–20. L. w. 1–24 + $\bigcirc$ cyl. + 1–72 ax. 90°=20–20. I had great difficulty for two months to persuade him to persist in the use of the glasses for all near work, but he finally became a thorough believer. He is now perfectly delighted with his glasses, and says that but for them he knows he would have lost his place. His vision now, with glasses, at distance is 20–20, though of course he only needs them for close use.

*Case 5.*—Miss W., age 18, in perfect health, though she frequently and severely suffers with headaches, especially whenever she engages in drawing or painting. Vision normal (20–20). With atropia, R. 20–100, L. 20–70. R. w. + 1–24 and L. w. + 1–30, vis.=20–20. She wrote me recently that her glasses had effected a perfect cure of her trouble. She had no trouble in using them from the first.

We must not overlook the fact that though there may be normal, or even more than normal acuteness of vision, yet there may co-exist serious errors of refraction, which, in order to see clearly for close distance, the patient must overcome by exerting to abnormal degree his muscle of accommodation.

*Case 6.*—Miss McR., age 18 or 20, music teacher. Came to me to be treated for granulated lids. She says she knows she is near-sighted, as indeed she really appears to be. She just wished her lids treated. She has no idea of using glasses. As I can find no trouble of the eyelids, it appears to me as if she is suffering from ciliary spasm. After a while she consents to a test of her refraction. Under atropia, her vison is R. 20–50, L. 20–70. R. and L. w. + 1–48=20–20. The use of these glasses soon gave her great relief, and as she expresses it, "they are her best friends."

*Case 7.*—I will show the importance of using a mydriatic in anything like peculiar cases of presbyopia. Mrs. W., age forty-eight, has "had trouble all her life with her eyes." She has never found any satisfactory glasses; vision, R. and L., 20–40, with atropia R=20–50, L=20–70, with +1–30, R=20=15, L=20–30. Her presbyopia is 1–20, and this added to her total hypermetropia (+1–30)=1–12. With + 1–12 glass she reads and sews in comfort; at her age, she of course supposed she would not need a 12 glass, and was very much surprised when I ordered it for her.

*Case 8.*—Shows how an uncorrected error of refraction may entail serious intra-ocular disease if not properly attended to. Mr. L., age thirty-two, (banker) is very closely occupied in his counting room. He came to me last January with a very serious and most obstinate iritis, especially in the right eye. Large masses of lymph in the aqueous and vitreous. Vision, R=20–200, L=20–30. Accepts—glass, but of course no special test of refraction was attempted. After a five months' tedious case of iritis he was discharged as cured. When carefully tested his refraction under atropia, was R. 20–70, L. 20–100, R. and L. w. + 1–30=20–15. For prudential reasons I did not give him the fully correcting glass, but ordered a + 1-36. With their use he has fully resumed his duties, and I have just had a letter stating that he considered himself fully relieved. In this case there was absolutely no other cause for his iritis than his uncorrected error of refraction.

*Case 9.*—Mr. C., (book-keeper) age twenty-eight, has had for two or three years a very troublesome conjunctivitis, and thinks of giving up his occupation. Refuses, however, to consent to the mydriatic, and goes home. In two months' time he returns and a test is made. His vision is R. and L. 20–20; with atropia, R. and L. 20–30; with + 1–60, R. and L. 20–15. It is remarkable, but true, that this slight error of refraction caused all his trouble. With these + 1–60 glasses he was almost immediately relieved of all his unpleasant symptoms.

*Case 10.*—Miss R., age 17. Three years ago I operated upon both internal recti for a strabismus of over three lines. The operation was successful. She had not been able to use her eyes at study to amount to anything. Her vision was R. 20–200, L. 20–40. Under atropia, w. + 1–18, R.=20–50; under atropia, w. + 1–24, L.=20–20. March 14th, last, I saw her and found her vision with glasses was the same as it was three years ago, and in spite of her amblyopia, she gets along very comfortably, with a moderate amount of study. She gets most comfort from the *constant* use of her glasses.

I think these three final cases should convince us of the impossibility of surely showing up the real error without the use of a mydriatic.

*Case 11.*—Mrs. R., age 25. Has had great trouble for several months with her eyes, aching of the balls and inability to read or sew. Vision, R. and L. 20-20. With atropia, R. and L. 20-200. It took three careful sittings of over an hour each to find the proper correction. At the first she accepted a + 1–18 glass, and appeared as if not astigmatic. But at the third one it was clear that the proper glass was R. w. + 1–24 $\bigcirc$ cyl. + 1–72 ax. 90°= 20–20. L. w. + 1–24 $\bigcirc$ cyl. + 1–48 ax. 90°=20–20. These glasses, while they worried her at first, soon relieved her of her trouble.

*Case 12.*—Mr. W., age 28. Theological student. Is trying to use a + 1–36 glass, which was prescribed for him by an eye specialist two years ago, but he is confident his glasses are wrong. Is unable to study with any degree of comfort. Vision, R. 20–20, L. 20–30. With atropia, R. and L. 20–40. R. w. + 1–72 ⊂⊃=cyl. 1–72 ax. 30°=20–20. L. w. + 1–48 ⊂⊃=cyl. 1–72 ax. 70°=20–20. These glasses have given him entire relief.

As to my own case, I had suffered for years with my eyes. More or less constant conjunctivitis, and severe headaches upon close use of my eyes. Ophthalmoscopic work had become especially trying. Even when at the theatre, or when I visited picture galleries, I would generally come away with severe headaches. I wished to feel that I was not ametropic, and supposed that my trouble was due to my rather delicate general health. Finally I saw that something must be done. I had gotten so I could not read a newspaper through, and I did not dare to read at night. As test of my eyes had not been made under atropia, I thoroughly atropinized one eye one week and the other the next week. By this means I could carry on my work with one eye. Under atropine, R. and L=20–100; R. and L. w. + 1–48⊂⊃ cyl. + 1–48, axis 980°=20–15. I also had a pair of cylindrical + 1–48, axis 90°, made for occasional use at distance. I frequently find these latter very beneficial to rest my eyes.

As to my fully correcting compound glasses, they are almost a revelation to me. I am quite sure that no one who has emmetropic eyes can appreciate the extreme degree of comfort which they have given me. I am frequently astonished—indeed I may say almost uneasy—at the amount of extra use to which I frequently put my eyes now. At first my fully correcting glasses gave me great trouble. They would cause a most unpleasant giddiness and, very frequently, aching in the temples, but by persisting in their use until I gradually became accustomed to relaxing my over-accommodation they soon gave me, as I said, great comfort. While I only use them for close work, my vision is with them now 20–10 easy.

In regard to the more recent (and very expensive) mydriatic, homatropine, the effect of which is so much more evanescent upon the accommodation than atropia, my experience is yet so recent with it that I am not prepared to give a valuable opinion concerning it. Usually I have found that after its full effect the accommodation is restored in thirty-six to forty-eight hours. It is almost amusing to note the difference in opinion as to its value, held by various eye surgeons. Recently two distinguished oculists, each surgeon to large eye hospitals in two different cities North, gave me their opinion of it. One claimed that it only partially paralyzed the accommodation and that he had abandoned its use. The other said it fully paralyzed the accommodation and that he used nothing else in refraction work. At the present writing my experience is that it does not generally *fully* paralyze the accommodation.

